# Endobronchial Hamartoma Presenting as Recurrent Pneumonia and Chronic Cough

**DOI:** 10.7759/cureus.13717

**Published:** 2021-03-05

**Authors:** Mihir Odak, Mohammed AlAzzawi, Abbas Alshami, Ghadier Alsaoudi, James Cosentino

**Affiliations:** 1 Internal Medicine, Jersey Shore University Medical Center, Neptune City, USA; 2 Pulmonary and Critical Care Medicine, Jersey Shore University Medical Center, Neptune, USA; 3 Pulmonary and Critical Care, Jersey Shore University Medical Center, Neptune, USA

**Keywords:** endobronchial hamartoma, community acquired pneumonia, bronchoscopy

## Abstract

Pneumonia is an infection of the lungs that can result from various etiologies, including bronchial obstruction. It is estimated that 5.4% of community-acquired pneumonia occurs as a result of an endobronchial obstruction, classifying them as post-obstructive pneumonia. Pulmonary hamartomas are benign and exceedingly rare tumors. These hamartomas are usually asymptomatic and found incidentally on imaging, however, they can cause patients to develop post-obstructive pneumonia.

We present a 40-year-old female with cough, fatigue, and recurrent right lower lobe pneumonia. Upon workup with bronchoscopy and biopsy, she was subsequently found to have an endobronchial hamartoma resulting in recurrent pneumonia in the same location. We are happy to report that the patient had a resection of the mass, as well as of the affected lung lobe, and has been pneumonia-free for five months. We hope to encourage a greater index of suspicion for endobronchial masses, including rare tumors, when a patient presents with recurrent pneumonia in the same location.

## Introduction

Hamartomas are benign tumors that are characterized by an uneven mixture of connective tissue, cartilage, muscle, fibrous tissue, and adipose tissue [1]. They are rare, solitary, benign pulmonary neoplasms, with an incidence of 0.025%, and are generally found incidentally on chest imaging [1-2]. Endobronchial hamartomas, however, account for approximately 5%-10% of all pulmonary hamartomas while the rest are present in the periphery of the lung field [1]. These masses can vary in size but have an average diameter of 4 cm [1]. Although hamartomas are benign and only mildly affect their surrounding tissues, they can cause severe symptoms, depending on their location. We present a case of endobronchial hamartoma that presented as recurrent pneumonia.

## Case presentation

A 40-year-old female with a past medical history significant for asthma, chronic obstructive pulmonary disease (COPD) due to 10-pack-year smoking history, and recurrent right lower lobe (RLL) pneumonia, presented with worsening cough and fatigue. The patient reported pleuritic chest pain localized to the right lower chest but denied shortness of breath, fever, chills, weight changes, nausea, symptoms of acid reflux, or difficulty swallowing. She had three right lower lobe pneumonias during the last eight months, with the most recent occurrence being in the previous six weeks when she was treated with cefuroxime and prednisone as an outpatient. She drinks alcohol occasionally and does not ingest or inject illicit drugs. She has no pertinent family history and is up to date with immunizations, including the pneumococcal polysaccharide PPV23 vaccine.

**Figure 1:**
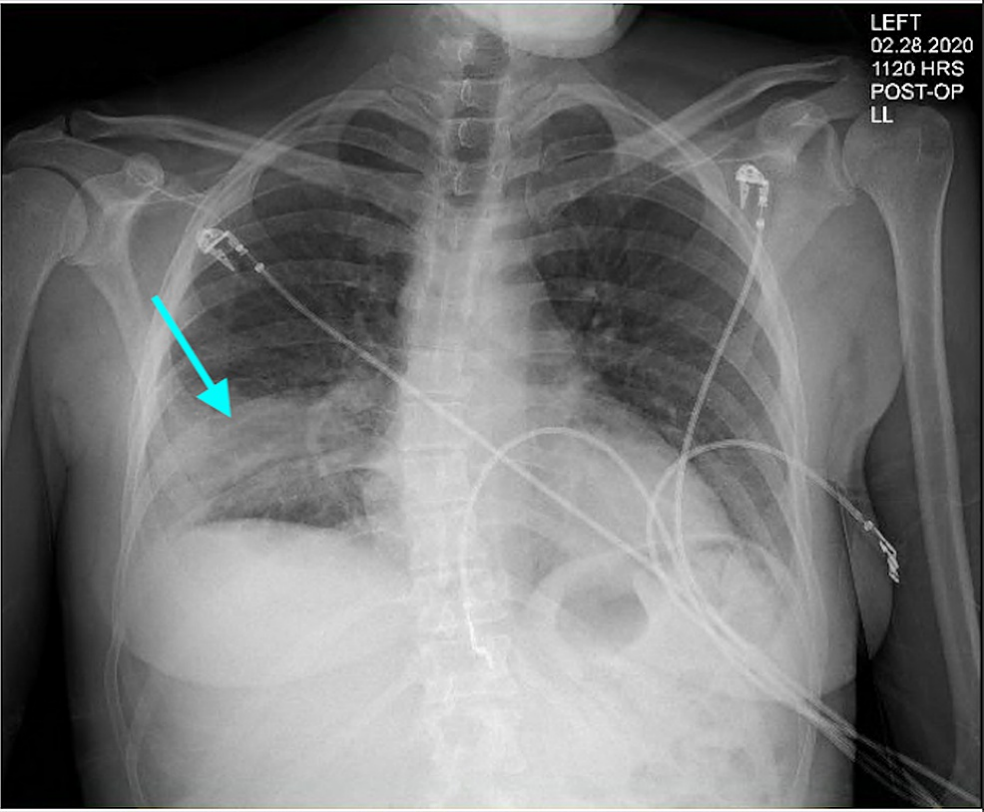
Chest X-ray showing right lower lobe lobar consolidation (blue arrow)

**Figure 2:**
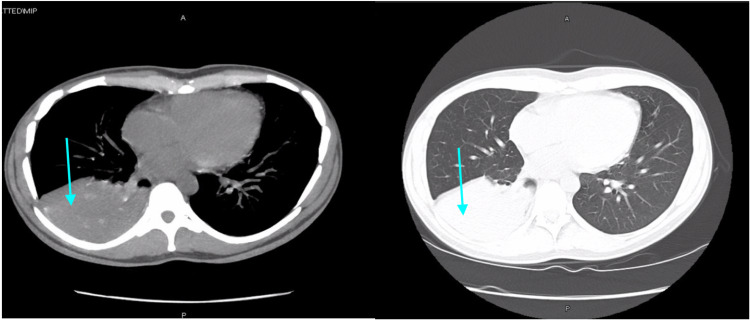
CT chest showing right lower lobe consolidation (blue arrows)

**Figure 3:**
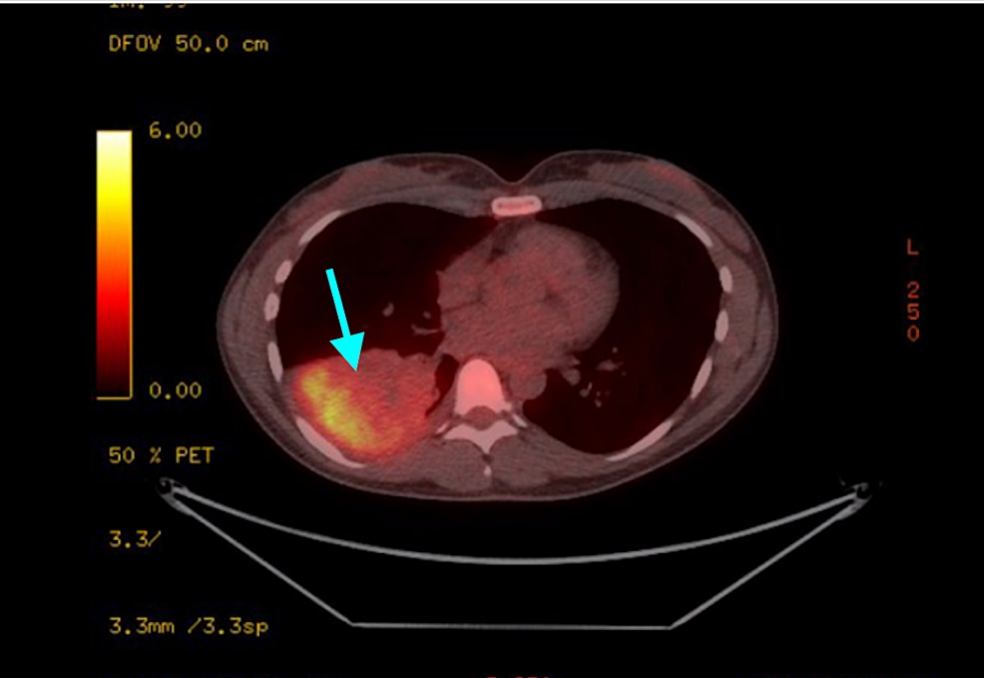
PET/CT imaging showing increased uptake in the right lower lobe (blue arrow) PET: positron emission tomography

**Figure 4:**
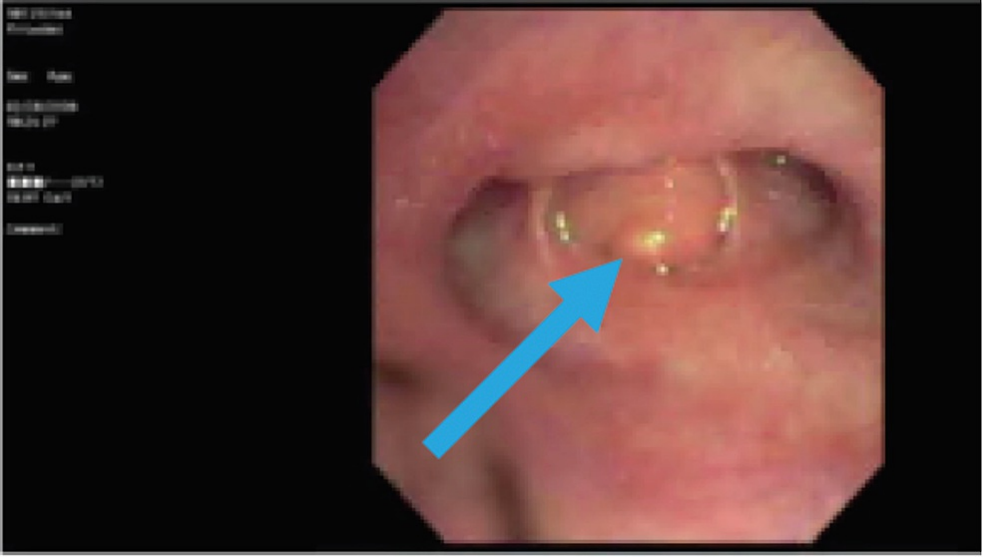
Bronchoscopy of right lower lobe segmentary bronchus showing an endobronchial mass at the opening of the segmentary bronchus (blue arrow)

On presentation, the patient’s vital signs were a blood pressure of 110/70 mmHg, a heart rate of 76 beats per minute, a temperature of 98.6 degrees Fahrenheit, respiratory rate of 18 breaths per minute, and oxygen saturation of 97%. The patient was in mild distress on physical exam, with an unremarkable cardiac exam. On pulmonary exam, the patient had decreased breath sounds in the right upper lobe (RUL) and RLL, and a normal left lung exam. She did not have rales; however, her exam was positive for mild wheezes in the right lower lung field. Chest X-ray showed right lower lung opacity (Figure 1), and computed tomography (CT) scan of the chest revealed an obstructing nodular opacity in the right lower lobe bronchus with post-obstructive RLL consolidation (Figure 2). This was confirmed with positron emission tomography (PET), which showed increased uptake in the RLL corresponding with a confluent infiltrate (Figure 3). The PET scan also revealed an obstructive nodule with post-obstructive pneumonia (Figure 3). Bronchoscopy revealed a large completely obstructing polypoid and vascular lesion in the right lower lobe (Figure 4). Fine needle biopsy revealed a nodular aggregate of bland appearing chondroid tissue with a lobulated contour, without significant cellular pleomorphism, binucleation, or mitotic activity. This confirmed a final diagnosis of an RLL endobronchial hamartoma. The patient underwent robotic video-assisted thoracoscopic surgery (VATS) for removal of the mass and right lower lobectomy. At her five-month follow-up appointment, she reported no further symptoms.

## Discussion

The origin of pulmonary hamartomas is thought to occur from mesenchymal stem cells that differentiate along the chondroid, smooth muscle, or adipose tissue cell lines [3-4]. As these tumors occur in the lung parenchyma, they do not actively cause any symptoms or discomfort to patients [5]. Their discovery may be incidental on chest imaging for another cause [3]. Our patient’s presentation is unique, as the hamartoma within her bronchi resulted in her symptoms of chronic cough and fatigue, complicated by recurrent pneumonia of the right lung. Parenchymal hamartomas constitute 90% of all pulmonary hamartoma cases while endobronchial hamartomas constitute only 10% [6] and contribute to the symptomatology of airway obstruction with recurrent respiratory infections and chronic cough [1].

Our patient was initially treated with a standard approach of treating pneumonia. As she fell in the low-risk group on the pneumonia severity index (PSI) and had no significant comorbidities, she was given outpatient treatment with levofloxacin 500 mg once daily for five days initially, followed by cefuroxime (recommended dose is 500 mg twice daily for five days [7]) and prednisone 20 mg with a five-day taper [8]. Her four recurrent cases of pneumonia, all being in the RLL, prompted further investigation, which showed an obstruction and post-obstructive consolidation on CT scan and PET scan. This obstructing tumor was likely causing obstructive pneumonia, due to the patient’s inability to clear secretions downstream of the obstruction, which would then get infected.

Recurrent pneumonia is characterized by either at least two episodes in one year or at least three episodes over an individual’s lifetime [9]. As our patient had four recurrences in a period of eight months, her condition can be designated as recurrent pneumonia. Possible etiologies of recurrent pneumonia include underlying pulmonary conditions such as COPD, immunologic deficiencies such as HIV/AIDS, and structural complications like mucus plugs or mass effects [10]. Our patient was not having COPD exacerbations at the time of symptoms onset and does not have any history of immunologic compromise. Recurrent pneumonia in the same region should raise suspicion for an endobronchial lesion, as masses within the bronchial tree can cause irritation to the walls and subsequently a cough. As an X-ray can be limited in revealing not only the cause of pneumonia but pneumonia itself, this suspicion should be followed up with a chest CT scan and bronchoscopy with biopsy if needed.

## Conclusions

The treatment of endobronchial hamartoma depends upon the severity of the case. In cases of asymptomatic endobronchial hamartomas, endoscopic excision or sleeve excision may be employed while in cases of recurrent pneumonia secondary to endobronchial hamartoma, a lobectomy may be required, as was required for our patient. The benefit of lobectomy in obstructing endobronchial hamartomas is complete resolution and prevention of recurrence, assuming the initial tumor was an isolated occurrence and not as a result of an underlying condition. Five months after the removal of her right lower lobe, our patient remains well and symptom-free. In this way, we hope to encourage the consideration of endobronchial masses, including rare tumors like hamartomas, when creating a differential diagnosis and evaluating patients with persistent cough and recurrent pneumonia.
